# Integrated genomic and transcriptomic approaches reveal oxidative stress adaptation mechanisms in a mesotrione‐resistant *Amaranthus tuberculatus* biotype

**DOI:** 10.1002/ps.70721

**Published:** 2026-03-11

**Authors:** Martin Laforest, Marianne Bessette, Geneviève Gagnon, Sandra Flores‐Mejia, Brahim Soufiane, Marie‐Claire Goulet, Dominique Michaud

**Affiliations:** ^1^ Agriculture and Agri‐Food Canada (AAFC), Saint‐Jean‐sur‐Richelieu Research and Development Centre St‐Jean‐sur‐Richelieu QC Canada; ^2^ Centre de recherche et d'innovation sur les végétaux, Département de phytologie Université Laval Québec QC Canada; ^3^ Centre de recherche sur les grains (CÉROM) Saint‐Mathieu‐de‐Beloeil QC Canada

**Keywords:** *Amaranthus tuberculatus*, waterhemp, herbicide resistance, mesotrione, GWAS, RNA‐seq, oxidative stress, nontarget‐site resistance

## Abstract

**BACKGROUND:**

*Amaranthus tuberculatus* (waterhemp) is a major agricultural weed known for its rapid evolution of resistance to multiple herbicide modes‐of‐action. Resistance to 4‐hydroxyphenylpyruvate dioxygenase (HPPD) inhibitors such as mesotrione is particularly concerning, yet the underlying genetic and molecular mechanisms remain incompletely understood. This study investigated the basis of mesotrione resistance in a Canadian waterhemp population (biotype 714) using an integrated genomic and transcriptomic approach.

**RESULTS:**

A genome‐wide association study (GWAS) on 152 individuals identified 11 unique single nucleotide polymorphisms (SNPs) significantly associated with resistance, with the most significant SNP located on chromosome 13 (*P* < 10^−26^). Gene Ontology (GO) enrichment of genes proximal to these SNPs implicated pathways related to electron transport and oxidoreductase activity. Transcriptomic analysis of treated plants revealed 187 differentially expressed genes (DEGs) between resistant and susceptible phenotypes. GO enrichment of these genes highlighted significant upregulation of pathways related to redox homeostasis, glutathione metabolism, lipid signaling and secondary metabolism. Weighted Gene Co‐expression Network Analysis (WGCNA) further corroborated these findings, identifying gene modules correlated with resistance that were enriched for functions in oxidative stress response and detoxification.

**CONCLUSION:**

Mesotrione resistance in this *A. tuberculatus* population is a complex, polygenic trait. Our findings indicate that resistance is not conferred by a single mechanism, but rather by an enhanced metabolic capacity to mitigate herbicide‐induced oxidative stress. This multifaceted defense involves the coordinated regulation of numerous detoxification, signaling, and metabolic pathways. Understanding this complex form of nontarget‐site resistance (NTSR) is critical for devising future management strategies to combat the spread of herbicide‐resistant weeds. © 2026 The Author(s). *Pest Management Science* published by John Wiley & Sons Ltd on behalf of Society of Chemical Industry.

## Graphical abstract

Integrated GWAS and transcriptomics in a Canadian waterhemp biotype reveal mesotrione resistance is polygenic and metabolically driven. Significant SNPs and 187 herbicide‐responsive genes point to enhanced redox homeostasis, glutathione‐linked detoxification, lipid/secondary metabolism, and oxidative stress responses. Resistance arises from coordinated, nontarget‐site metabolic defenses mitigating HPPD inhibitor damage.

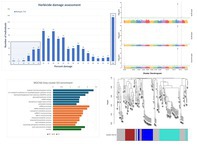

## 1 INTRODUCTION

*Amaranthus tuberculatus* (Moq.) Sauer, 1955 is a member of the Amaranthaceae family.^1^ All dioecious species of the genus *Amaranthus*, including *A. tuberculatus* and *Amaranthus palmeri* S. Watson (1877) belong to the subgenus *Acnida* and are native to North America.^2^ As a C4 plant, *A. tuberculatus* has a high photosynthetic rate allowing it to rapidly produce plants up to 2–3 m tall, making it highly competitive with crops.^2^ It is heat‐tolerant and can survive–even thrive–under low‐light conditions. The species produces very small pollen grains, favoring long‐distance gene flow via wind dispersal.^1^ A single plant can produce ≤2.3 million seeds, effectively creating a large seedbank.^3^ Similar to pollen, these seeds can disperse over long distances via water, farm machinery, manure and compost, wind, birds and other animals.^1^ Remarkably, viability rate and gut retention times in migratory waterfowl indicate a potential dispersal distance of ≤2900 km from the source.^4^ Additionally, these seeds have a late and extended emergence period during the cropping season.^5^ Cultural practices increasingly adopted since the 1980s, including reduced tillage, have further favored small‐seeded weed species such as *A. tuberculatus*.^3^ Collectively, these characteristics make *A. tuberculatus* a formidable crop competitor and one of the most problematic weeds in the Midwestern United States.^6^

No‐till, and to a large extent conservation tillage, allows farmers to efficiently manage larger land areas while minimizing energy, labor and machinery costs. At the same time, it serves as an effective soil conservation practice, improving water and fertilizer utilization and often resulting in higher yields compared to conventional tillage.^7^ This technique, however, requires effective weed control strategies. Its adoption in corn coincided with the introduction of atrazine, followed by other active ingredients such as 2,4‐d, dicamba and paraquat.^7^ The introduction of glyphosate and glyphosate‐resistant crops further simplified weed control in no‐till systems and contributed to the widespread popularity of this practice.^8^

In this context, *A. tuberculatus* increasingly colonized cropped areas and was exposed to selection pressure from multiple herbicides. Unsurprisingly, it has evolved resistance to numerous modes‐of‐action (MoAs) and is particularly adept at adaptation. Among dicots, it ranks second only to *A. palmeri*, another dioecious amaranth, in the number of herbicide groups to which resistance has developed. Although *A. palmeri* is resistant to 11 different MoA groups, *A. tuberculatus* follows closely with resistance to seven, and possibly eight, groups.^9^, ^10^ These two species are considered the most economically important weeds owing to both their wide distribution and the lack of effective herbicide options for their control, given their resistance to many MoAs.^9^, ^11^

Waterhemp has evolved resistance to 4‐hydroxyphenylpyruvate dioxygenase (HPPD) inhibitors and several studies have sought to elucidate the underlying mechanisms and inheritance patterns. Ma *et al*.^12^ showed that malathion caused a moderate increase in mesotrione activity compared to the herbicide alone, suggesting the involvement of an unidentified cytochrome P450 monooxygenase. Huffman *et al*.^13^ demonstrated a complex inheritance pattern of the resistance trait: more plants than expected survived the low rate herbicide whereas the opposite happened at higher dose. Kaundun *et al*.,^14^ studying a Nebraska population, identified 4‐hydroxylation and, most importantly, impaired translocation as resistance mechanisms.

In 2019, a population of suspected mesotrione‐resistant *A. tuberculatus* was collected in Québec, Canada. The field is believed to have been contaminated via second‐hand machinery originating from the United States,^15^ a situation reminiscent of the first case in Ontario in 2002 (Mike Cowbrough, pers. comm.,^16^, ^17^). The objective of this study was to confirm mesotrione resistance in this population and investigate potential resistance mechanisms through transcriptional analysis and genome‐wide association.

## 2 MATERIALS AND METHODS

### 2.1 Origin of the Amaranthus mesotrione‐resistant population

In Québec, the first waterhemp outbreak was identified at a farm located in Montérégie‐Ouest in the fall of 2017.^15^ The population suspected of mesotrione resistance (biotype 714) was collected in the region of Lanaudière, Québec, Canada in 2019. Mesotrione had not been applied in the collection year or during the four preceding years.

### 2.2 Herbicide treatment

Herbicide treatments were performed using the commercial product Callisto 480™ (Syngenta, Basel, Switzerland). The recommended label rate (1×) is 0.21 L ha^−1^ [100.8 g active ingredient (a.i.) ha^−1^], and the corresponding double dose (2×) is 0.42 L ha^−1^ (201.6 g a.i. ha^−1^). The optimum spray stage for waterhemp control is when plants have fewer than eight leaves.^18^ A nonionic surfactant, Agral 90™ (Syngenta), was added to all treatments–including the 0× control–at 0.25% v/v. Spray volume was 200 L ha^−1^, applied with a Teejet flat‐fan nozzle (XR 80015VS). For a genome‐wide association study (GWAS), resistance and damage assessment were evaluated according to the scale developed previously.^19^

### 2.3 Genotyping by sequencing

Libraries for triple digest GbS (3D‐GBS) were prepared following the protocol of Poland *et al*.^20^ with modifications, at the Plateforme d'Analyses Génomiques of the Institut de Biologie Intégrative et des Systèmes (IBIS, Université Laval, Québec, Canada). Modifications included the use of three restriction enzymes (*Pst*I/*Nsi*I/*Msp*I) instead of the usual dual *Pst*I/*Msp*I combination, and the use of a BluePippin system (SAGE sciences, Beverly, ME, USA) to size libraries before PCR amplification (elution set between 50 and 65 min on a 2% gel). Plate barcodes were added according to Colston‐Nepali *et al*.^21^ Libraries were loaded onto an AVITI instrument (Element Biosciences, San Diego, CA, USA) using a 2 ×75 Cloudbreak FS kit to generate SE150 reads, following the manufacturer's instructions.

### 2.4 Sequence analyses and single nucleotide polymorphism (SNP) genotyping

Sequences were first demultiplexed using ea‐utils 1.1.2.779 fastq‐multx,^22^ followed by quality assessment and trimming with fastp 0.24.0.^23^ Reads were mapped to AmaTu_v01, the *A. tuberculatus* reference genome obtained from the International Weeds Genomics Consortium database (https://weedpedia.weedgenomics.org/), using BWA 0.7.17‐r1188.^24^ Read alignments were sorted, indexed with samtools 1.10, and polymorphisms were identified using bcftools 1.14.^25^, ^26^ Further polymorphism filtering was carried out with vcftools 0.1.16 using the following parameters: max‐missing 0.5, mac 3, minQ 30, minDP 3).^27^ Individuals with >50% missing data were removed.

### 2.5 Calculation of genetic distances, UPGMA clustering, GWAS analysis

Genotype data were converted to the *genind* class using the *vcfR2genind* function from the poppr package,^28^ enabling distance calculations and multivariate analyses. Pairwise genetic distances among individuals were calculated using the bitwise genetic distance implemented in *bitwise.dist* (poppr package), which is well‐suited for biallelic SNP data and provides a robust measure of genetic similarity based on allele sharing.^28^ A hierarchical agglomerative clustering analysis was performed with the unweighted pair group method with arithmetic mean (UPGMA) algorithm on the distance matrix, using the *hclust* function with method set to ‘average’. The generated dendrogram was converted to a *phylo* object with the *as.phylo* function from the ape package,^29^ for advanced visualization and further processing. The dendrogram was visualized using the ggtree package^30^ for enhanced customization and annotation within the ggplot2 ecosystem.^31^ Group membership was indicated by coloring tip labels according to the sample group. GWAS analysis were performed in R^32^ using the vcfr 1.15.0,^33^ ggplot2 3.5.1^31^ and GAPIT 3.4.0^34^ packages. The models BLINK (Bayesian‐information and Linkage‐disequilibrium iteratively Nested Keyway), MLMM (Multi‐Locus Mixed Model) and FarmCPU (Fixed and Random model Circulating Process Unifying) were used to calculated genome‐wide associations, with a minor allele frequency threshold of 0.05. Linkage blocks were defined using the solid spine method in haploview 4.2.^35^

### 2.6 Gene annotation, gene ontology enrichment and weighted correlation network analyses

All genes identified in the genome were annotated with interproscan 5.75–106.^36^ The *‐‐goterms* function was used to assign Gene Ontology (GO) terms to annotated gene, linking protein signatures to standardized functional categories. GO enrichment analysis was performed using the topgo package^37^ in R. Statistical significance was determined with Fisher's exact test, comparing observed *versus* expected counts of genes annotated with each GO term. To control for the multiple testing, *P*‐values were adjusted using the Benjamini–Hochberg procedure to adjust for false discovery rate (FDR). GO terms with adjusted *P*‐values <0.05 were considered significantly enriched. These terms represent the biological functions, molecular processes and cellular components that are over‐represented in the input set of genes compared to the background set. Results were visualized with bar plots, where the x‐axis represents GO terms and the y‐axis shows the −log_10_ (*P*‐value). Weighted gene co‐expression network analysis (WGCNA) was performed using the R package WGCNA 1.73^38^ with a power of 28 for scale‐free topology and a minimum module size of 25.

### 2.7 RNAseq data analysis

Leaves from sprayed plants (described above) of biotype 714 were collected 24 h after treatment. Total RNA was extracted from individual plants that survived herbicide application (resistant) and from those that died 28 days after treatment (susceptible), using a RNeasy Mini Kit (Qiagen, Germantown, MD, USA). RNASeq libraries were prepared and sequenced at the Génome Québec Centre d'expertise et de Service (Montréal, QC, Canada) with an Illumina polyA‐enriched RNA library kit (Illumina, San Diego, CA, USA). Sequencing generated 100 bp paired‐end reads on an Illumina NovaSeq platform. Data quality was assessed and adapter/quality trimming performed with fastp.^23^ Reads were aligned to the *A uberculatus* genome (same as above) with hisat2 ^39^ and gene‐level counts were generated with htseq‐count.^40^ Differential expression analysis was performed using the R package TCC,^41^ with tmm normalization, edger as the test method, three iterations and a false discovery rate (FDR) cutoff of 0.1. Genes with a Benjamini–Hochberg adjusted *q*‐value ≤0.05 were considered significantly differentially expressed.

## 3 RESULTS

### 3.1 Origin of the mesotrione resistant biotype

The material used in this study was a population of waterhemp (*Amaranthus tuberculatus*) that exhibited resistance to the herbicide mesotrione. Resistant plants were originally identified and selected from field sites where mesotrione had been applied previously. In an effort to study the inheritance of resistance, selected resistant individuals were crossed with susceptible ones to generate a segregating populations. Despite repeated attempts, these crosses produced very few progeny, resulting in a limited number of plants available for further analysis. The difficulties encountered in generating and maintaining this population are likely to reflect the genetic complexity of mesotrione resistance in waterhemp. As a result, the material used for this research consisted of seeds originally collected in the field.

### 3.2 Phenotypic assessment

**Figure 1:**
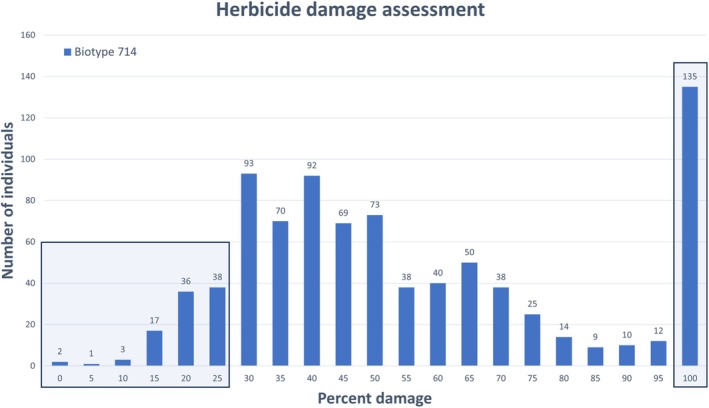
Damage assessment of biotype 714 after mesotrione application, according to the Brown and Farmer scale.^19^ Visual inspection of treated plants was done 6 weeks after treatment. The shaded boxes indicate the categories (≤ 25% and 100%) from which the samples were selected for the GWAS study.

A total of 865 plants from population 714 were tested for resistance to mesotrione at 100.6 g ha^−1^ (label rate), and damage was recorded (Fig. 1) according to the Brown and Farmer scale.^19^ Based on this evaluation, plants were selected for genotyping‐by‐sequencing: 97 plants showing ≤25% damage were selected to represent resistant individuals, and 70 individuals with 100% damage also were selected to represent susceptible individuals for biotype 714.

### 3.3 Genotyping and population structure

A total of 5 373 097 SNPs were identified after alignment to the *A. tuberculatus* genome (IWGC; Eric Patterson, pers. comm.). After quality filtering, this number was reduced to 373 458 SNPs. Of the 158 individuals, 152 were retained when excluding samples with >50% missing data, yielding 85 resistant and 67 susceptible individuals. An UPGMA genetic distance tree was generated using these markers (Supporting information Fig. S1). Samples did not cluster according to phenotype: both susceptible individuals and those resistant to mesotrione were found throughout the clusters. Likewise, the genetic distance between individuals of the population as calculated with the VanRaden method was used to create a kinship matrix for the individuals of the resistant population (Fig. S2). Again, there was no apparent structure in the population, the additive genetic relatedness for most individuals being centered around zero.

### 3.4 Genome‐wide association study

**Figure 2:**
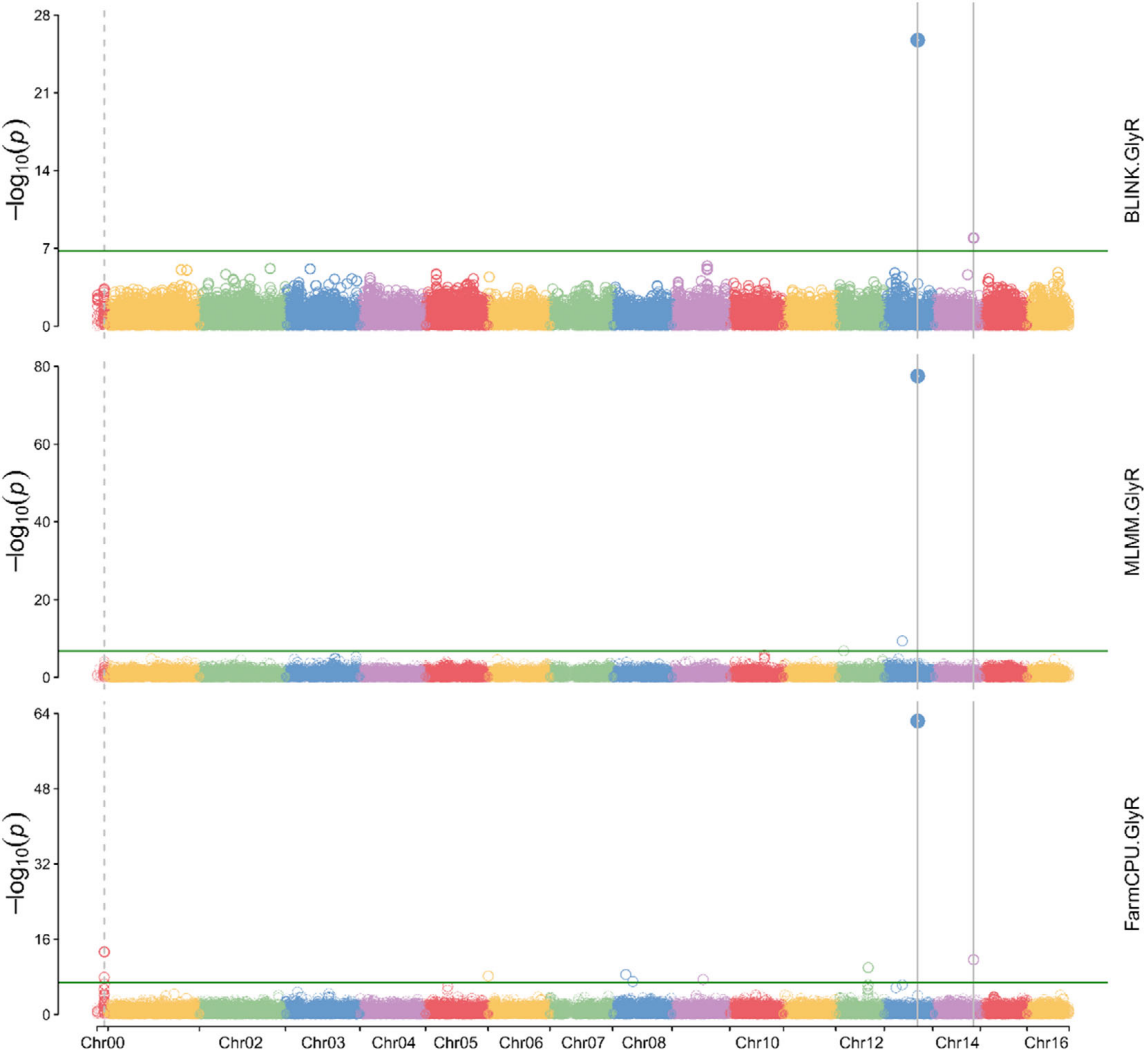
Manhattan plots showing genome‐wide associations between SNP markers and the resistance phenotype in population 714. The three panels correspond to BLINK, MLMM and FarmCPU models. The x‐axis corresponds to positions on each A. tuberculatus genome and the y‐axis is the –log10 of the *P*‐value. A green line depicts the Bonferroni‐corrected significance threshold.

A GWAS was performed between SNP markers and the resistance trait using three models: BLINK, MLMM and FarmCPU (Fig. 2). All models identified significant associations with the resistance trait. The most stringent model, BLINK, identified two SNPs associated with survival to mesotrione. MLMM and FarmCPU highlighted three and nine SNPs, respectively. Quantile‐quantile (Q‐Q) plots indicated that population structure, or the lack thereof, did not influence the results, as most probabilities matched expectations (Fig. S3). The SNP with the lowest *P*‐value across all three models was located on chromosome 13 at position 21 037 430 (Table 1). A second SNP, on chromosome 14 at position 25 693 063, was identified by both BLINK and FarmCPU. MLMM identified two additional SNPs associated with resistance (Chr12_4 941 960, Chr13_11 306 035). FarmCPU identified a further seven SNPs (Chr00_4 555 459, Chr00_4 605 586, Chr06_69 381, Chr08_8 328 744, Chr08_12 751 902, Chr09_19 488 907 and Chr12_20 355 278).

**Table 1:** SNPs associated with mesotrione resistance in population 714 including *P*‐values with associated minor allele frequencies (MAF), model used to identify each SNP, phenotype variance explained in percentages, linkage blocks limits as define by haploview and genes contained in the blocks

SNP	Chr	Pos	*P*‐value	MAF	Model	(%)	Linkage blocks limits	Genes in linkage blocks	Annotation
Chr00_4 555 459	Chr00	4 555 459	1.20E−08	0.26	armCPU	0	4 547 311‐4 566 017	AmaTu_RefChr00g253110 AmaTu_RefChr00g253170 AmaTu_RefChr00g253350 AmaTu_RefChr00g253150 AmaTu_RefChr00g253080	Cytochrome b/b6/petB 60Sribosomal protein L11 related ‐ ‐ NADH:quinone oxidoreductase/Mrp antiporter, TM
Chr00_4 605 586	Chr00	4 605 586	4.72E−14	0.25	FarmCPU	6.14E‐06	4 592 744‐4 607 503	AmaTu_RefChr00g253370 AmaTu_RefChr00g253420 AmaTu_RefChr00g253430 AmaTu_RefChr00g253440 AmaTu_RefChr00g253380 AmaTu_RefChr00g253410 AmaTu_RefChr00g253400 AmaTu_RefChr00g253360	Ycf1 Transmembrane protein Poly(A) RNA polymerase, mitochondrial‐like, central palm domain ‐ ‐ Tetraspanin family NADH‐quinone oxidoreductase, subunit D Pol polyprotein, beta‐barrel domain
Chr06_69 381	Chr06	69 381	6.19E−09	0.21	FarmCPU	0	69 371‐69 435		
Chr08_8 328 744	Chr08	8 328 744	3.10E−09	0.41	FarmCPU	0.29	8 328 739‐8 423 811		
Chr08_12 751 902	Chr08	12 751 902	9.19E−08	0.16	FarmCPU	1.96E‐06	12 751 898‐12 751 915		
Chr09_19 488 907	Chr09	19 488 907	3.47E−08	0.29	FarmCPU	1.22		AmaTu_RefChr09g161700	PWI domain
Chr12_4 941 960	Chr12	4 941 960	1.46E−07	0.09	MLMM	11.64	4 924 113‐4 941 983		
Chr12_20 355 278	Chr12	20 355 278	1.04E−10	0.19	FarmCPU	6.01E‐06	20 355 238‐20 370 911	AmaTu_RefChr12g193740 AmaTu_RefChr12g193750	Transmembrane protein Ribosomal L18 C‐terminal region
Chr13_11 306 035	Chr13	11 306 035	4.19E−10	0.49	MLMM	16.96	11 306 033‐11 306 036		
Chr13_21 037 430	Chr13	21 037 430	1.77E−26	0.41	BLINK	88.56	20 980 775‐21 037 511	AmaTu_RefChr13g209310 AmaTu_RefChr13g209330 AmaTu_RefChr13g209320 AmaTu_RefChr13g209380 AmaTu_RefChr13g209370 AmaTu_RefChr13g209350	Phospholipase D Active site motif Membrane‐associated salt‐inducible protein‐like Homeobox protein transcription factor KNOX1 domain (transcription factor) Molybdopterin cofactor sulfurase MOSC Major facilitator superfamily
Chr13_21 037 430	Chr13	21 037 430	2.79E−78	0.41	MLMM	66.73			
Chr13_21 037 430	Chr13	21 037 430	4.08E−63	0.41	FarmCPU	0			
Chr14_25 693 063	Chr14	25 693 063	1.13E−08	0.21	BLINK	2.89	25 685 850‐25 693 180	AmaTu_RefChr14g225160 AmaTu_RefChr14g225220	CLMP1 family MEMO1 family
Chr14_25 693 063	Chr14	25 693 063	2.15E−12	0.21	FarmCPU	48.39			

**Figure 3:**
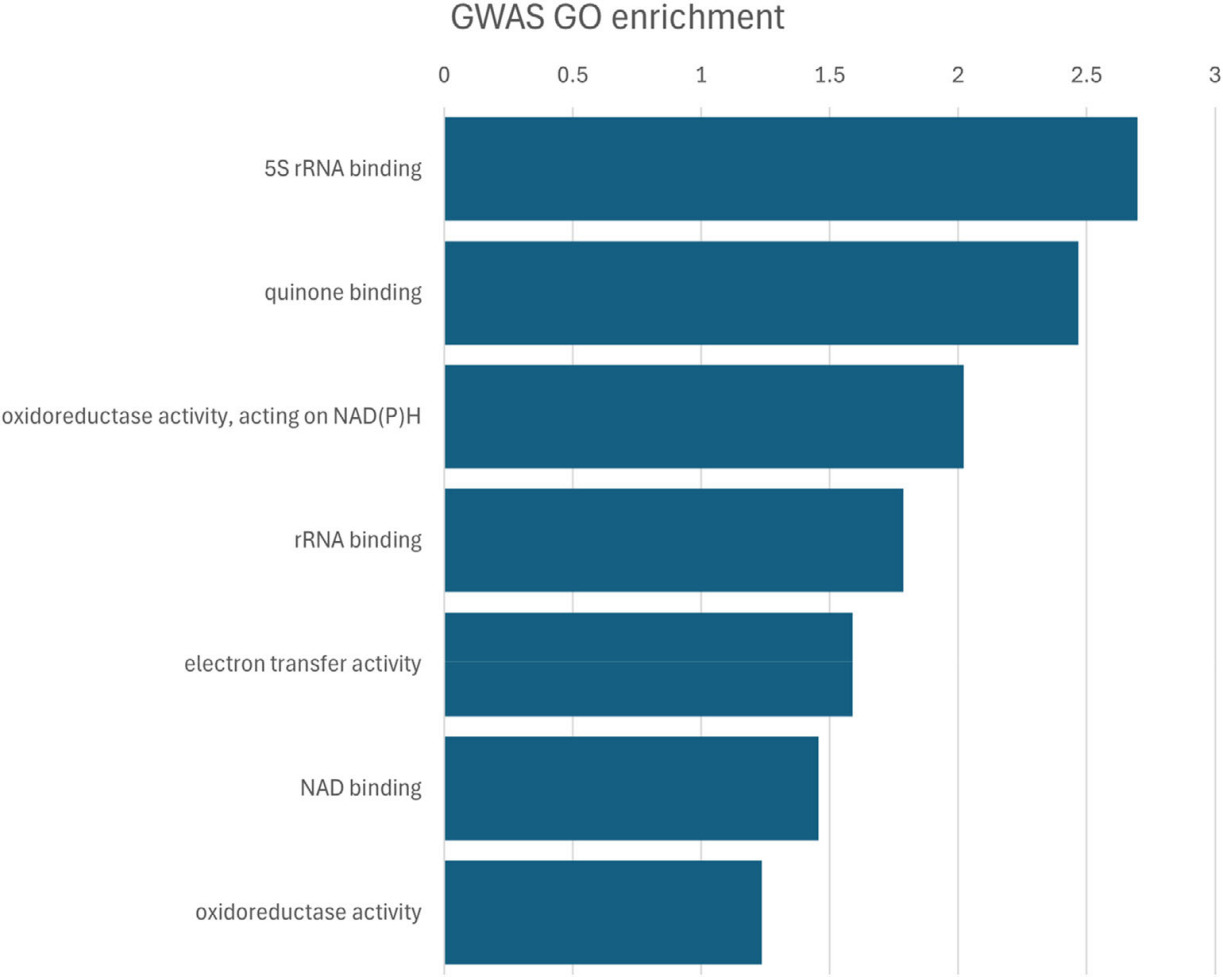
Gene Ontology terms associated with genes proximal to GWAS‐identified SNPs. Terms are categorized by molecular function (MF); no significant biological process (BP) or cellular component (CC) terms were found. The x‐axis is the –log_10_ of the *P*‐value.

The GO enrichment analysis was performed on the 23 genes located closest to the GWAS SNPs (Fig. 3; Table S1), in the linkage blocks defined in haploview. Aside from a more transcriptionally active response 5S RNA binding, the most significant molecular functions were related to electron transport, including quinones, oxidoreductase activity and NAD binding. This enrichment might be biased by the number of genes in the vicinity of GWAS hits in the mitochondrial DNA (13 of 23 genes identified are mitochondrial).

### 3.5 Identification and analysis of differentially expressed genes

Eight plants of biotype 714 (four resistant and four susceptible) were used for the differential gene expression experiment, which identified 187 genes (Table S2). The gene with the strongest signal was *AmaTu_RefChr13g213230*, annotated as the N‐terminal domain of ribose‐phosphate pyrophosphokinase. This gene is involved in the conversion of ribose 5‐phosphate into phosphoribosyl pyrophosphate (PRPP). Its expression was completely suppressed in resistant plants. Another gene, *AmaTu_RefChr03g063250*, annotated as a cofactor for the assembly of complex C, subunit B in the thylakoid membrane was overexpressed. *AmaTu_RefChr15g239390*, involved in thiamine (Vitamin B1) biosynthesis, and *AmaTu_RefChr04g080550*, encoding malonyl‐CoA decarboxylase (regulating malonyl‐CoA levels and lipid oxidation, also were overexpressed in resistant plants.

**Figure 4:**
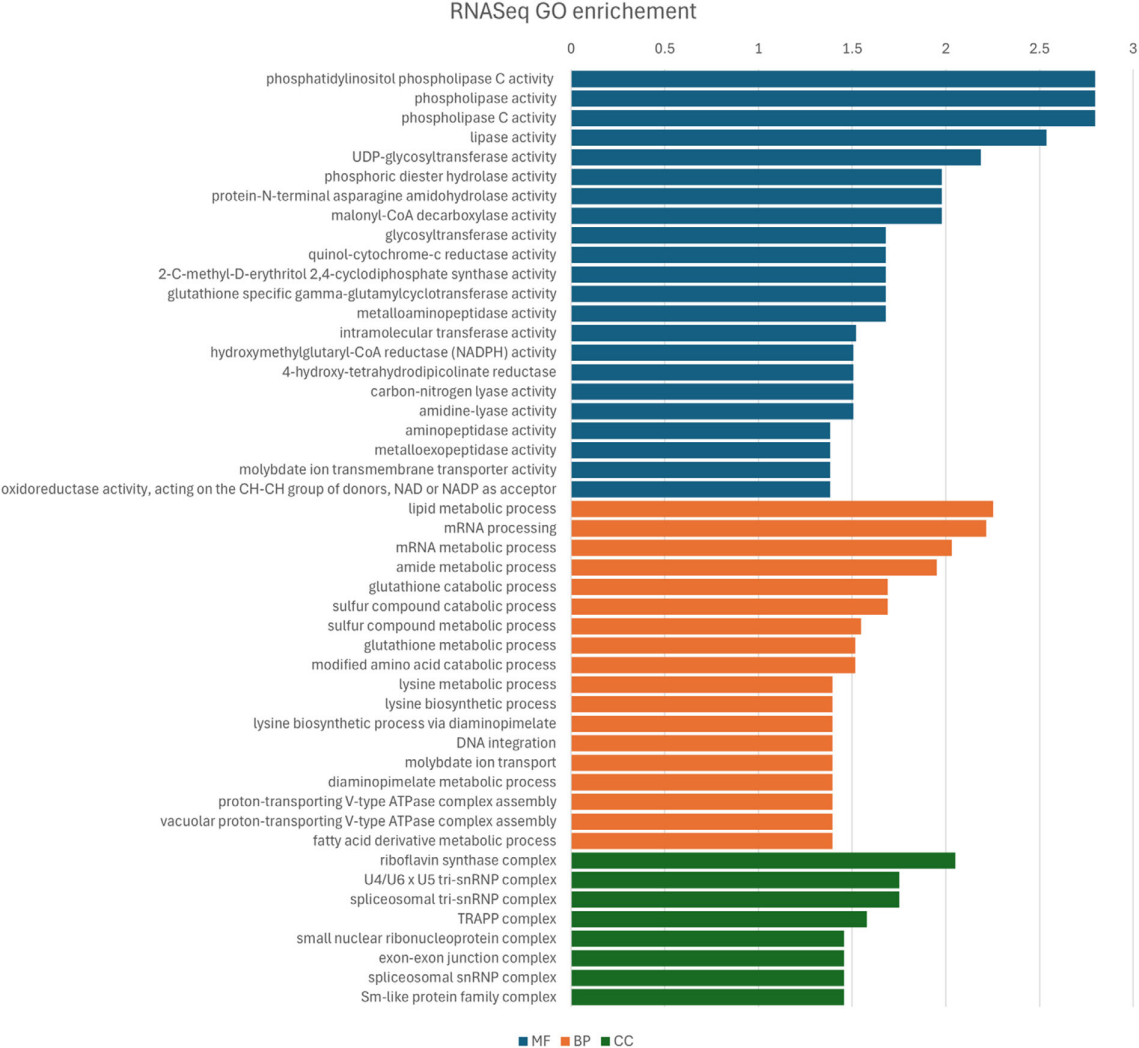
GO terms associated with DEGs. Terms are categorized by molecular function (MF), biological process (BP) and cellular component (CC). The x‐axis is the –log_10_ of the *P*‐value.

The GO enrichment analysis of the DEGs provided additional insights into resistance mechanisms (Fig. 4). Representative molecular functions included phospholipase, glycosyltransferase and malonyl‐CoA decarboxylase activities. Pathways involving mevalonate and isoprenoids were indicated by enrichment of 2‐C‐methyl‐d‐erythritol 2,4‐cyclodiphosphate synthase activity. Additionally, several redox homeostasis‐related pathways were identified, including hydrolyzation, quinol‐cytochrome‐c reductase, hydroxymethylglutaryl‐CoA reductase (NADPH), 4‐hydroxy‐tetrahydrodipicolinate reductase, and oxidoreductase activity involving CH‐CH donors with NAD/NADP acceptors. Furthermore, the analysis highlighted nitrogen and molybdenum involvement in response to mesotrione. These enriched molecular functions are reflected in the identified biological processes, such as lipid and fatty acid metabolism, amide and amino acid metabolism, and pathways related to glutathione, sulfur compounds and lysine. Proton‐transporting V‐type ATPases, known to be redox‐regulated, and the riboflavin synthase complex also were identified.^42^

**Figure 5:**
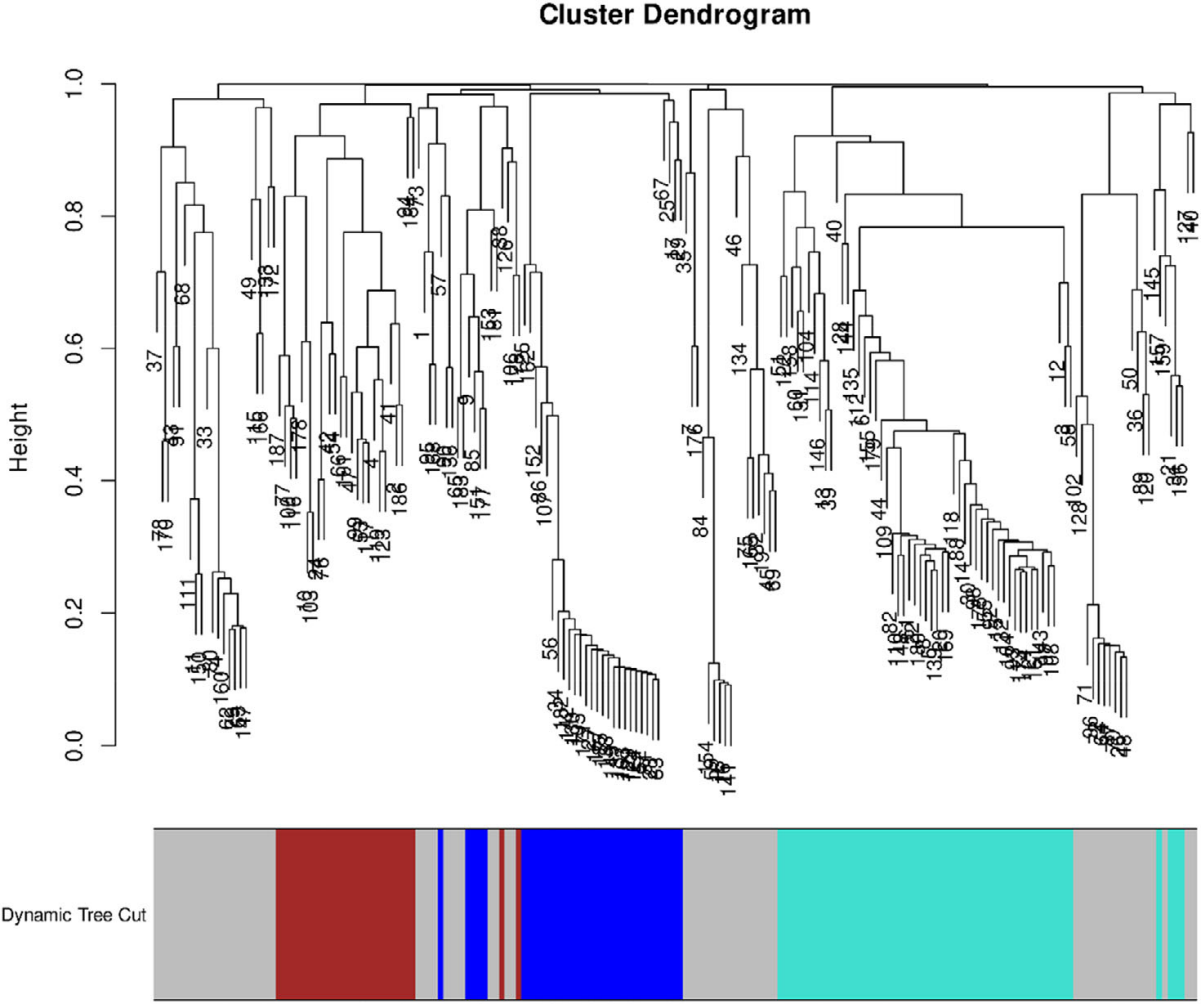
Hierarchical clustering dendrogram derived from WGCNA of gene expression profiles. Each branch represents a set of co‐expressed genes, partitioned into four modules (clusters) indicated by unique colors beneath the branches. Modules correspond to groups of genes potentially sharing functional relationships or regulatory mechanisms. Module assignment was determined using dynamic tree cut parameters.

**Figure 6:**
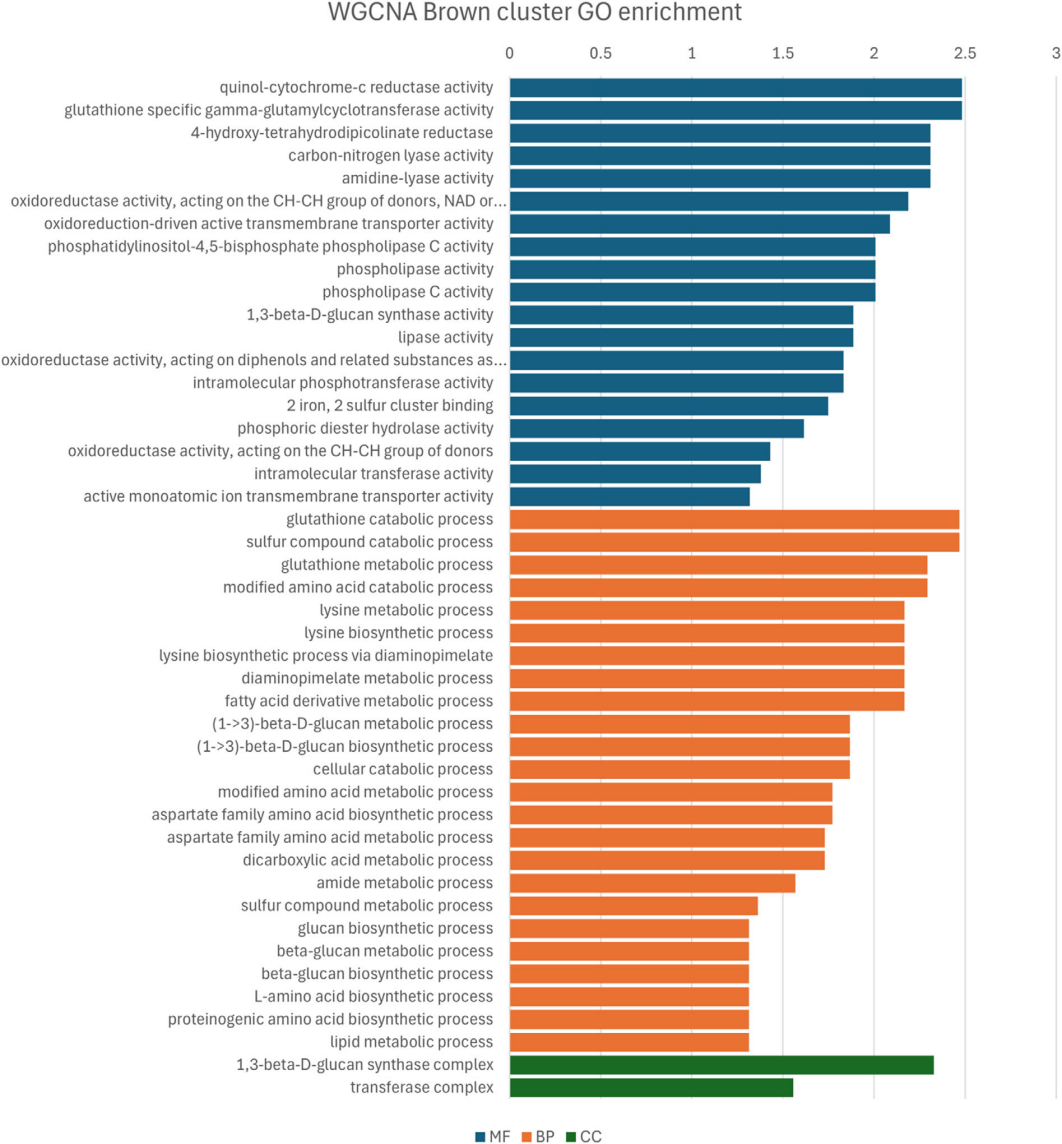
GO enrichment analysis of genes in the ‘brown’ WGCNA modules. Top enriched GO terms (Fisher >0.05) are shown for molecular function (MF), biological process (BP) and cellular component (CC). The x‐axis is the –log_10_ of the *P*‐value.

**Figure 7:**
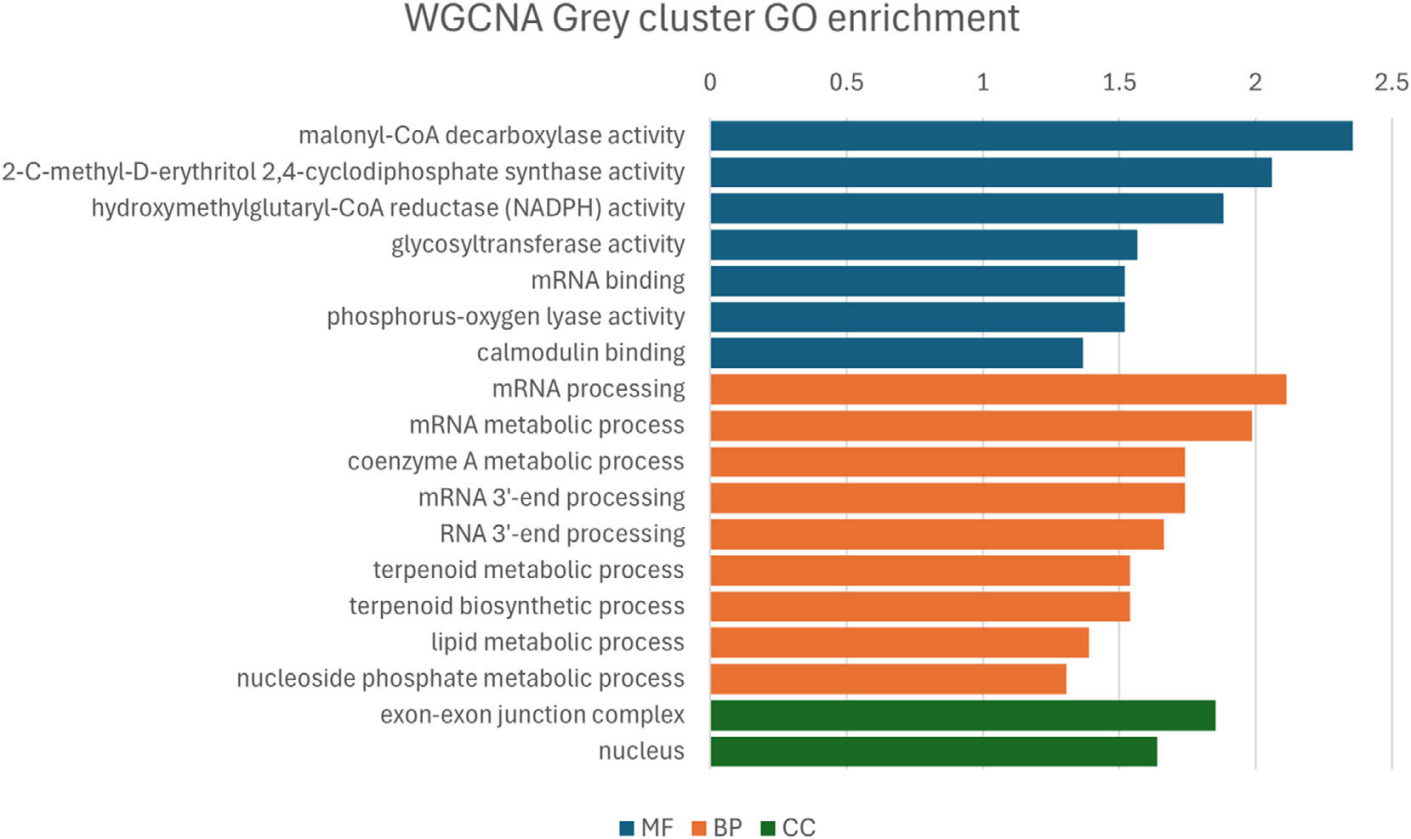
GO enrichment analysis of genes in the ‘grey’ WGCNA modules. Top enriched GO terms (Fisher >0.05) are shown for molecular function (MF), biological process (BP) and cellular component (CC) categories. The x‐axis is the –log_10_ of the *P*‐value.

**Figure 8:**
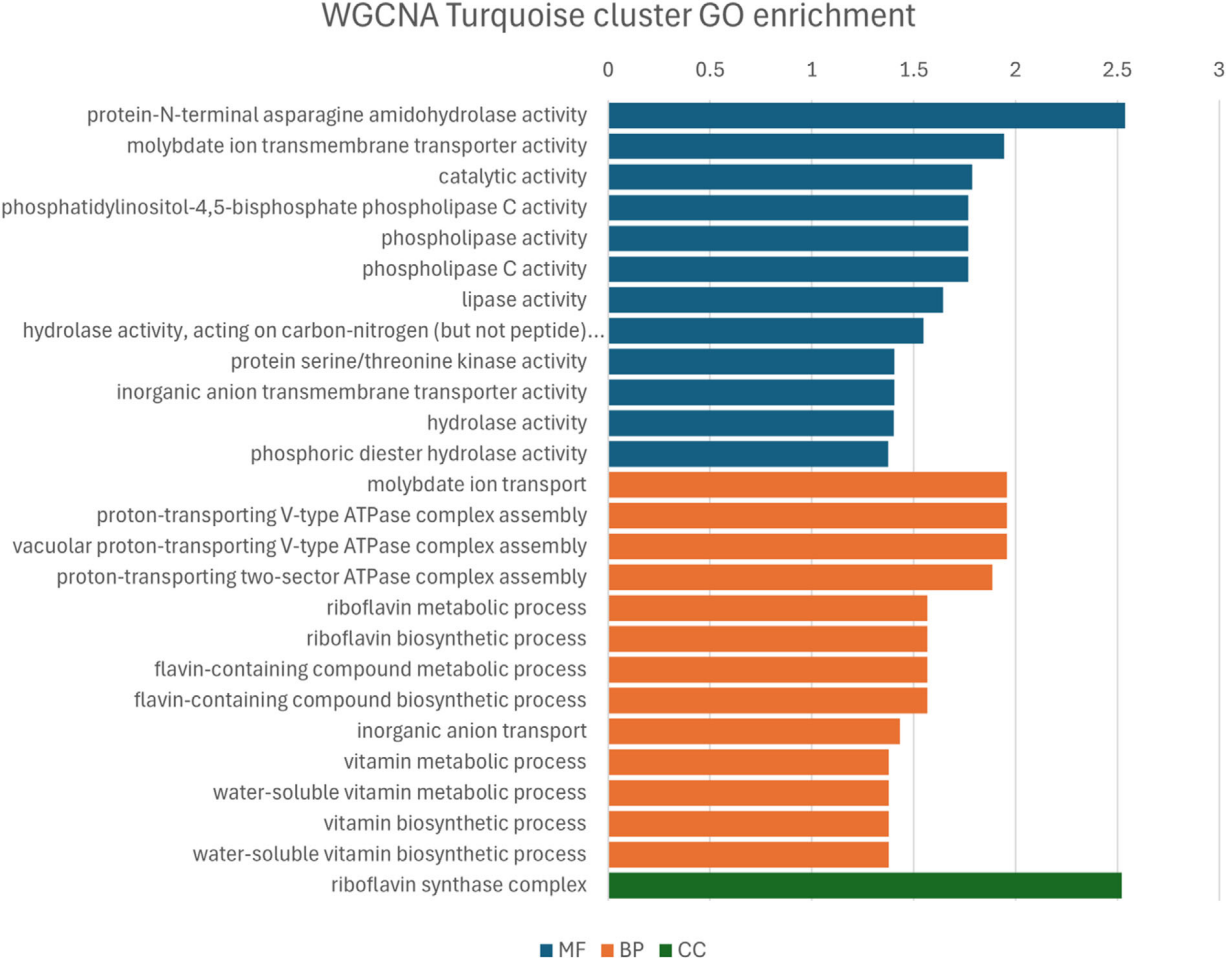
GO enrichment analysis of genes in the ‘turquoise’ WGCNA modules. Top enriched GO (Fisher >0.05) are shown for molecular function (MF), biological process (BP) and cellular component (CC) categories. The x‐axis is the –log_10_ of the *P*‐value.

To complement GO enrichment results, a WGCNA was performed using the 187 DEGs. This analysis aimed to uncover additional complexity in the gene regulatory networks underlying resistance. Resistant and susceptible lines grouped into distinct clusters based on gene expression (Fig. S4). Four modules were identified based on the similarity of their expression patterns among the eight individuals analyzed (Fig. 5). Three of these modules showed expression patterns correlating (threshold >0.7, *P*‐values shown in Fig. S5) with the resistance phenotype, clusters ‘brown’ (correlation = 0.73), ‘grey’ (correlation = 0.91) and ‘turquoise’ (correlation = −0.72), which comprise 27, 69 and 57 genes, respectively (Table S8). GO enrichment was performed on the gene lists of each of these clusters and the results are shown in Figs 6, 7, 8 (Tables S3–S5). Although the ‘grey’ and ‘brown’ modules were positively correlated, the ‘turquoise’ module was negatively correlated, indicating that, on average, genes in this last cluster have lower expression in the resistant biotypes.

The WGCNA results largely reflected those from differential expression analysis, because both were based on the same gene set, but helped disentangle certain aspects of the herbicide response. For example, glutathione activity, oxidative response and phospholipase activity were enriched in the ‘brown’ module, whereas malonyl‐CoA and transfer of glycosyl seemed to be enriched in the ‘grey’ module. Interestingly, the ‘brown’ module also included biological processes involving lysine and aspartate, whereas the ‘grey’ module was associated with mRNA processing, terpenoids and lipids. The ‘turquoise’ module, which was negatively correlated with resistance, encompassed phospholipase C activity and molybdate transport. Proton transport also emerged as a significant biological process in this module, along with pathways involving vitamin B12 (riboflavin) and flavin.

## 4 DISCUSSION

Resistance to 4‐hydroxyphenylpyruvate dioxygenase inhibitors has been investigated several times and the mechanism remains elusive. We initiated a study of this phenomenon in a biotype found in Quebec, Canada, with the aim of increasing knowledge by combining a GWAS with the characterization of differentially expressed genes. Both approaches identified distinct genes involved in several cellular activities. Interestingly, several biological processes were highlighted by both technologies. For example, both GWAS and RNASeq provided evidence for the involvement of redox state maintenance and oxidative stress adaptation in resistant plants compared with susceptible ones. Mesotrione‐resistant waterhemp appears to modulate its redox metabolism, which is likely to increase its capacity for detoxification of ROS, to which several biological processes contribute. First, the most differentially expressed gene (*AmaTu_RefChr13g213230*) encodes the N‐terminal domain of ribose phosphate pyrophosphokinase, an enzyme that converts ribose 5‐phosphate into 5‐*O*‐phosphono‐ribose 1‐diphosphate (PRPP), a precursor for nucleotide and NADP^+^ biosynthesis. By blocking ribose phosphate pyrophosphokinase, the pentose phosphate pathway (PPP) and its connection to glycolysis are favored, producing both NADPH and energy (ATP).^43^, ^44^ NADPH provides reducing power to scavenge ROS generated by herbicide action.

Additionally, regulation of genes involved in flavin and riboflavin biosynthesis ensures the supply of key cofactors for redox homeostasis, potentially enabling the plant to withstand oxidative stress.^45^ Molybdenum cofactor enzymes (e.g. nitrate reductase, sulfite oxidase) also may contribute to redox and nitrogen/sulfur metabolism under stress.^46^ Enhanced molybdate transport therefore supports these enzymes. Isoprenoid metabolism (including carotenoids and tocopherols) is also activated. Because carotenoids, whose synthesis is directly blocked by HPPD inhibitors, are essential antioxidants, the plant may attempt to compensate for their loss by boosting other related compounds for protection.^47^

The SNPs on chromosome 00 (Chr00_4 555 459 & Chr00_4 605 586) were unexpected, as they are located in the mitochondrial genome, which is generally thought to be maternally inherited and genetically identical within an individual. However, the long‐standing notion of clonality in mitochondrial genetics has been challenged by several studies in recent years.^48^, ^49^, ^50^ Genes proximal to these SNPs, as well as other genes encoding mitochondrion‐localized proteins (e.g. *AmaTu_RefChr12g194570* & *AmaTu_RefChr11g187870*), may indicate involvement of this organelle in cellular redox homeostasis during herbicide stress, possibly through pathways involving NADPH and glutathione.^51^ Glutathione metabolism and catabolism are likely to be preferentially implicated in detoxifying ROS rather than in conjugating the herbicide. Upregulated glutathione pathway genes indicate an active defense against oxidative and chemical stress. Interestingly, differentially expressed glutathione transferases (*AmaTu_RefChr02g034440* & *AmaTu_RefChr03g067320*) showed higher expression in susceptible individuals. Riboflavin‐derived coenzymes can function either as oxidizing or reducing agents (accepting or donating electrons) depending on the cellular context, and their redox behavior may vary unpredictably under specific stress conditions.^51^ Sulfur metabolism, another enriched biological process, is important for glutathione and many defense metabolites. Catabolism or adaptive metabolism here is likely to increase sulfur availability for glutathione and related compounds.^51^

There was also enrichment in phospholipase C activity. Phospholipases are key to lipid signaling and membrane remodeling. Upon mesotrione exposure and oxidative stress, activation of phospholipases may help signal stress, remodel damaged membranes and release lipid‐derived secondary messengers (e.g. inositol phosphates, diacylglycerol).^52^ Malonyl‐CoA, another activity identified by GO enrichment, is an important intermediate for fatty acid and secondary metabolite biosynthesis. The gene *AmaTu_RefChr04g080550*, annotated as malonyl‐CoA decarboxylase N‐terminal domain, catalyzes the conversion of malonyl‐CoA into acetyl‐CoA. The formation of Malonyl‐CoA is the first committed step in lipid biosynthesis. It also is an inhibitor of carnitine palmitoyltransferase I, which is responsible for fatty acid uptake into mitochondria for subsequent β‐oxidation.^53^ Because malonyl‐CoA decarboxylase depletes malonyl‐CoA, lipid transport into mitochondria and lipid oxidation increases, supplying more reducing equivalents (NADH, FADH₂) for the electron transport chain.^54^

The SNP located on chromosome 13, near position 21 037 430, accounts for the majority of the variation—≤88% as indicated by the BLINK model. This SNP is situated within a linkage block that includes six genes: a phospholipase d active site motif, a membrane‐associated salt‐inducible protein‐like gene, a homeobox protein transcription factor, a gene containing a KNOX1 domain (transcription factor), a molybdopterin cofactor sulfurase (MOSC) and a member of the major facilitator superfamily. Based on the available data, it is not possible to determine which of these genes may be involved in resistance. Likewise, the two genes within the linkage block on chromosome 14, which explain ≤48% of the variation according to the FarmCPU model (Table 1), were annotated using InterPro. AmaTu_RefChr14g225160 belongs to the CLMP1 family, a group of proteins involved in plastid separation in *Arabidopsis*.^55^ The second gene, AmaTu_RefChr14g225220‐mRNA‐1, encodes a protein with similarity to MEMO1 (mediator of ErbB2‐driven cell motility 1). Although little is known about MEMO1 in plants, studies in animals suggest it binds copper and protects against redox activity *in vitro*.^56^, ^57^ Unfortunately, further confirmation of the involvement of these two loci was not possible owing to challenges in maintaining the resistant population.

None of the overexpressed genes were found to be colocated with the resistance‐associated SNPs or their corresponding linkage blocks. This could suggest that the number of SNPs was insufficient to cover all linkage blocks in the genome, possibly owing to rapid LD decay. The high phenotype variance explained (PVE) by these SNPs, together with expression profiles pointing to different genes, suggests that while there are important elements within the linkage blocks on chromosomes 13 and 14 that contribute to resistance, these alone may not be sufficient to confer resistance.

Taken together, our findings illustrate that mesotrione‐resistant waterhemp deploys multiple overlapping and potentially complementary mechanisms to survive herbicide application. It is important to recognize, however, that not all resistance mechanisms may be required simultaneously in every plant. Within a population, individual plants may rely on distinct combinations of metabolic, transport, signaling and stress‐response strategies to overcome the effects of mesotrione. This diversity in adaptive strategies greatly increases the resilience of the population, but also complicates genetic analysis, making it challenging to pinpoint universal genetic determinants of resistance. Thus, the genetic architecture underlying resistance is likely to be heterogeneous both within and among populations, underlining the need for multifaceted investigative approaches to fully unravel this complex trait. These findings align with and extend previous research on HPPD‐inhibitor resistance in *Amaranthus* and other weed species, underscoring the complexity and diversity of nontarget site resistance mechanisms. Studies in waterhemp and Palmer amaranth have repeatedly implicated enhanced detoxification pathways, regulation of antioxidant metabolism, and metabolic plasticity as central contributors to resistance.^12^, ^58^ The identification of mitochondrial SNPs and upregulation of genes associated with redox and organellar metabolism in our study suggests the involvement of broader cellular networks, echoing emerging themes in recent literature.^59^ Our integrative GWAS and transcriptomic approach highlights novel candidate genes and pathways—such as lipid signaling and malonyl‐CoA metabolism—not prioritized previously. This reinforces the need for multifaceted investigations and functional studies of newly identified loci, as well as for management strategies that consider the evolutionary potential and metabolic flexibility of weedy populations.

## 5 CONCLUSION

Elucidating NTSR is of pivotal importance, as it may lead to alternative targets that could be exploited to mitigate the evolution of herbicide resistance. For example, resistance to HPPD inhibitors through increased tolerance to oxidative stress might be overcome by combining the herbicide with another ROS‐eliciting compound to completely overwhelm weed‐specific cellular mechanisms. Despite the inherent limitations of genotyping‐by‐sequencing, such as the rapid decay of linkage disequilibrium, and the constraints of RNAseq, where key causative genes may not always exhibit differential expression, our integrated approach successfully uncovered crucial cellular processes associated with mesotrione resistance in waterhemp. It is possible that some important resistance effectors may have been overlooked, yet our findings provide valuable insights into the underlying mechanisms and establish a strong foundation for future research aimed at achieving a more complete understanding of herbicide resistance in this species. In turn, this knowledge can be used to develop diagnostic tools for the early detection of herbicide resistance, thereby informing producers and helping them adapt control practices to better protect their crops.

## CONFLICT OF INTEREST

The authors declare no conflict of interest.

## Supporting information


**Figure S1.** Pairwise distance tree of population 714 calculated using the UPGMA method. Susceptible individuals are labeled in blue, and resistant individuals in red.
**Figure S2.** Kinship matrix among individuals of population 714 calculated using the VanRaden method. Cell color represents the estimated genomic relationship (kinship coefficient) between each pair of individuals, with lighter colors indicating higher relatedness and warmer colors indicating lower or negative kinship values. Diagonal values represent self‐kinship (inbreeding coefficients). The VanRaden method estimates additive genomic relationships based on marker genotypes. Susceptible individuals are labeled in blue, and resistant individuals are red.
**Figure S3.** Quantile‐quantile (QQ) plot of observed *versus* expected −log₁₀(p) values from GWAS. Each point represents a SNP. The diagonal line represents the null hypothesis of no association (expected distribution). Upward deviation in the upper‐right section indicates an excess of small *P*‐values, reflecting true marker‐trait associations or potential confounding effects, such as population structure.
**Figure S4.** Hierarchical clustering dendrogram of individuals based on gene expression data.
**Figure S5.** Heatmap depicting correlations between WGCNA‐identified gene modules (y‐axis) and external traits (x‐axis). Each cell displays the correlation coefficient and corresponding *P*‐value. Blue indicates negative correlation, white indicates no correlation, and red indicates positive correlation. Strong and significant correlations suggest biologically relevant modules.

**Table S1.** Results of gene ontology enrichment analysis for the genes identified by the GWAS.
**Table S2.** Results of gene ontology enrichment analysis for the genes identified by differential gene expression.
**Table S3.** Results of the gene ontology enrichment analysis for the genes identified by WGCNA, brown cluster.
**Table S4.** Results of gene ontology enrichment analysis for the genes identified by WGCNA, grey cluster.
**Table S5.** Results of gene ontology enrichment analysis for the genes identified by WGCNA, turquoise cluster.
**Table S6.** Expression data expressed as normalized counts for each sample.
**Table S7.** Differential expression analysis gene annotations.
**Table S8.** Genes identified in WGCNA, brown, grey and turquoise clusters.

## Data Availability

The data that support the findings of this study are available from the corresponding author upon reasonable request.
