# Supplementary material for: Integrated genomic and transcriptomic approaches reveal oxidative stress adaptation mechanisms in a mesotrione‐resistant Amaranthus tuberculatus biotype
Source: Pest Manag Sci. 2026 Mar 11;82(7):6387–98. doi: 10.1002/ps.70721 (PMC13240694; doi:10.1002/ps.70721)
Supplement: Supplementary file 1 — Figure S1. Pairwise distance tree of population 714 calculated using the UPGMA method. Susceptible individuals are labeled in blue, and resistant individuals in red. Figure S2. Kinship matrix among individuals of population 714 calculated using the VanRaden method. Cell color represents the estimated genomic relationship (kinship coefficient) between each pair of individuals, with lighter colors indicating higher relatedness and warmer colors indicating lower or negative kinship values. Diagonal values represent self‐kinship (inbreeding coefficients). The VanRaden method estimates additive genomic relationships based on marker genotypes. Susceptible individuals are labeled in blue, and resistant individuals are red. Figure S3. Quantile‐quantile (QQ) plot of observed versus expected −log₁₀(p) values from GWAS. Each point represents a SNP. The diagonal line represents the null hypothesis of no association (expected distribution). Upward deviation in the upper‐right section indicates an excess of small P‐values, reflecting true marker‐trait associations or potential confounding effects, such as population structure. Figure S4. Hierarchical clustering dendrogram of individuals based on gene expression data. Figure S5. Heatmap depicting correlations between WGCNA‐identified gene modules (y‐axis) and external traits (x‐axis). Each cell displays the correlation coefficient and corresponding P‐value. Blue indicates negative correlation, white indicates no correlation, and red indicates positive correlation. Strong and significant correlations suggest biologically relevant modules. [file PS-82-6387-s002.docx]

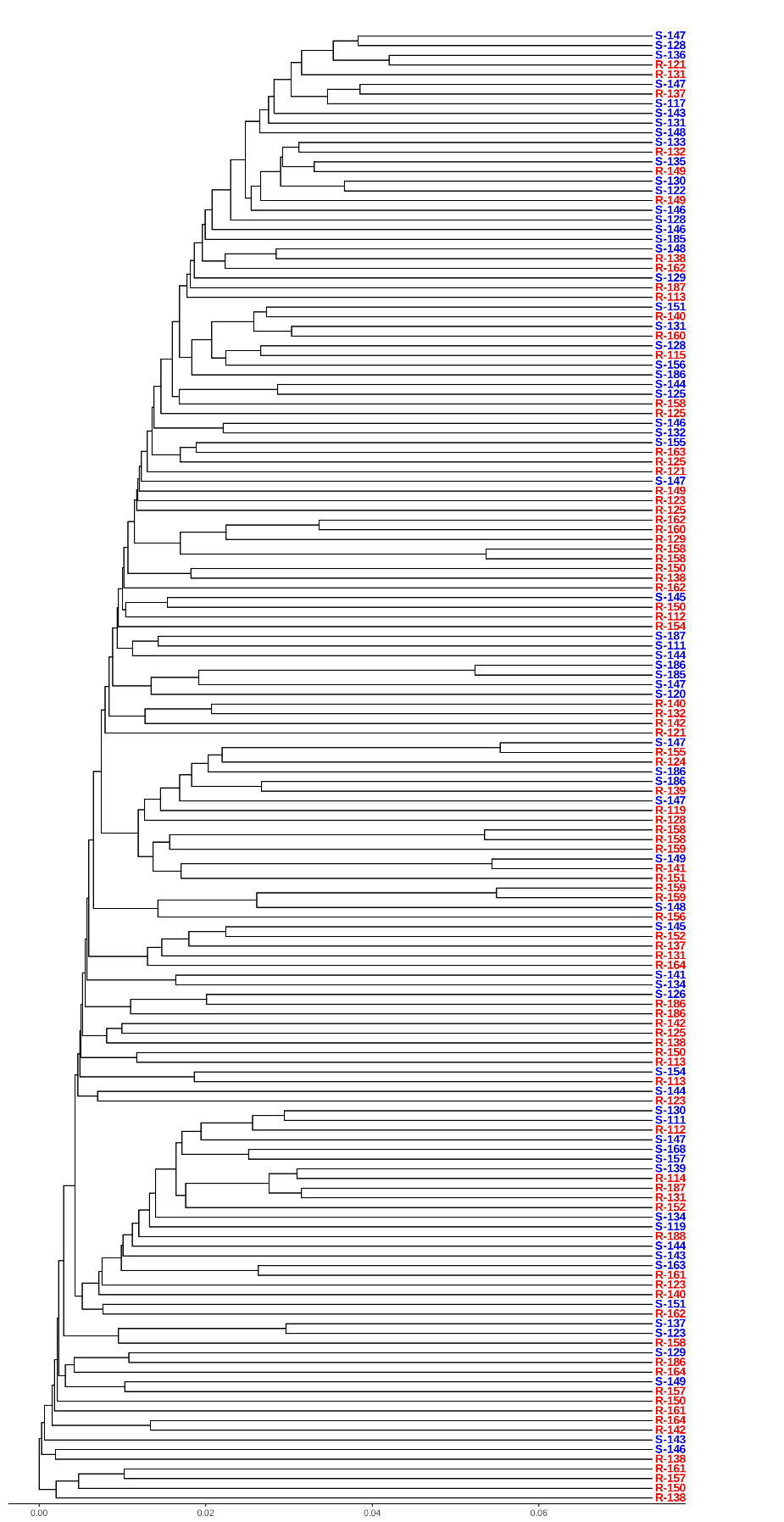


Supplementary Figure 1. Pairwise distance tree of population 714 calculated using the UPGMA method. Susceptible individuals are labeled in blue, and resistant individuals in red.


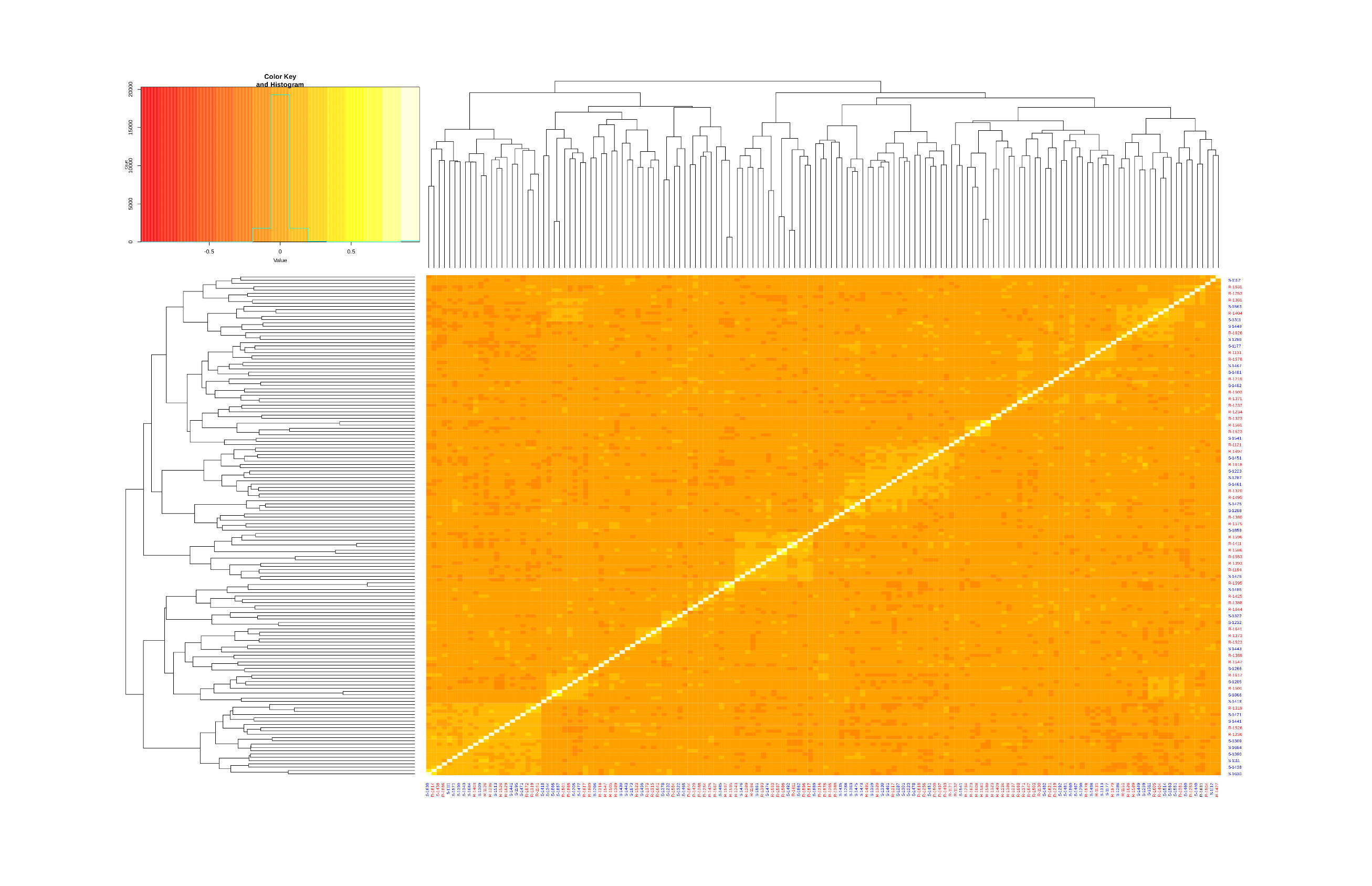


Supplementary Figure 2. Kinship matrix among individuals of population 714 calculated using the VanRaden method. Cell color represents the estimated genomic relationship (kinship coefficient) between each pair of individuals, with lighter colors indicating higher relatedness and warmer colors indicating lower or negative kinship values. Diagonal values represent self-kinship (inbreeding coefficients). The VanRaden method estimates additive genomic relationships based on marker genotypes. Susceptible individuals are labeled in blue, and resistant individuals are red.


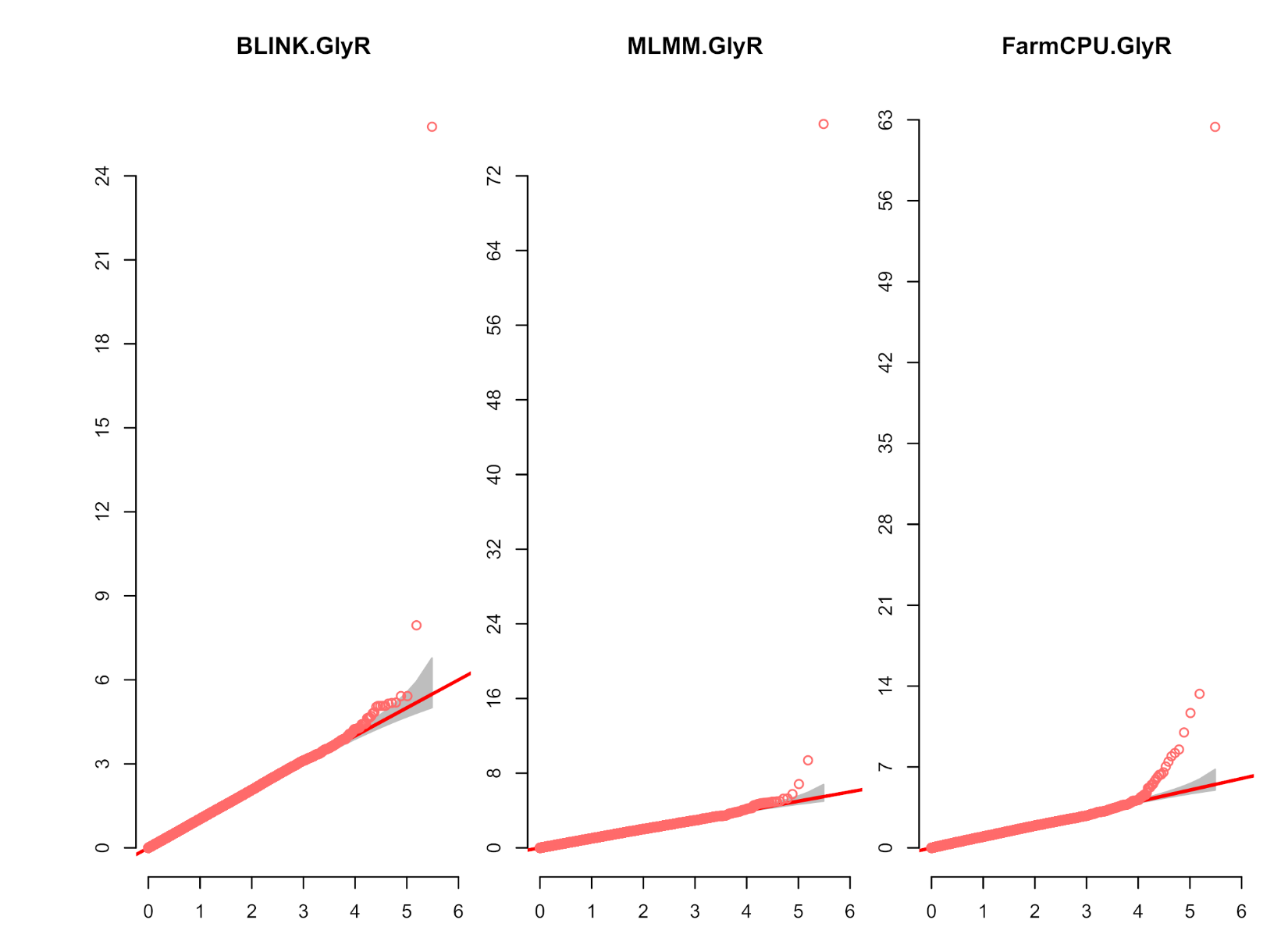


Supplementary Figure 3. Quantile-quantile (QQ) plot of observed versus expected −log₁₀(p) values from GWAS. Each point represents a SNP. The diagonal line represents the null hypothesis of no association (expected distribution). Upward deviation in the upper-right section indicates an excess of small p-values, reflecting true marker-trait associations or potential confounding effects, such as population structure.


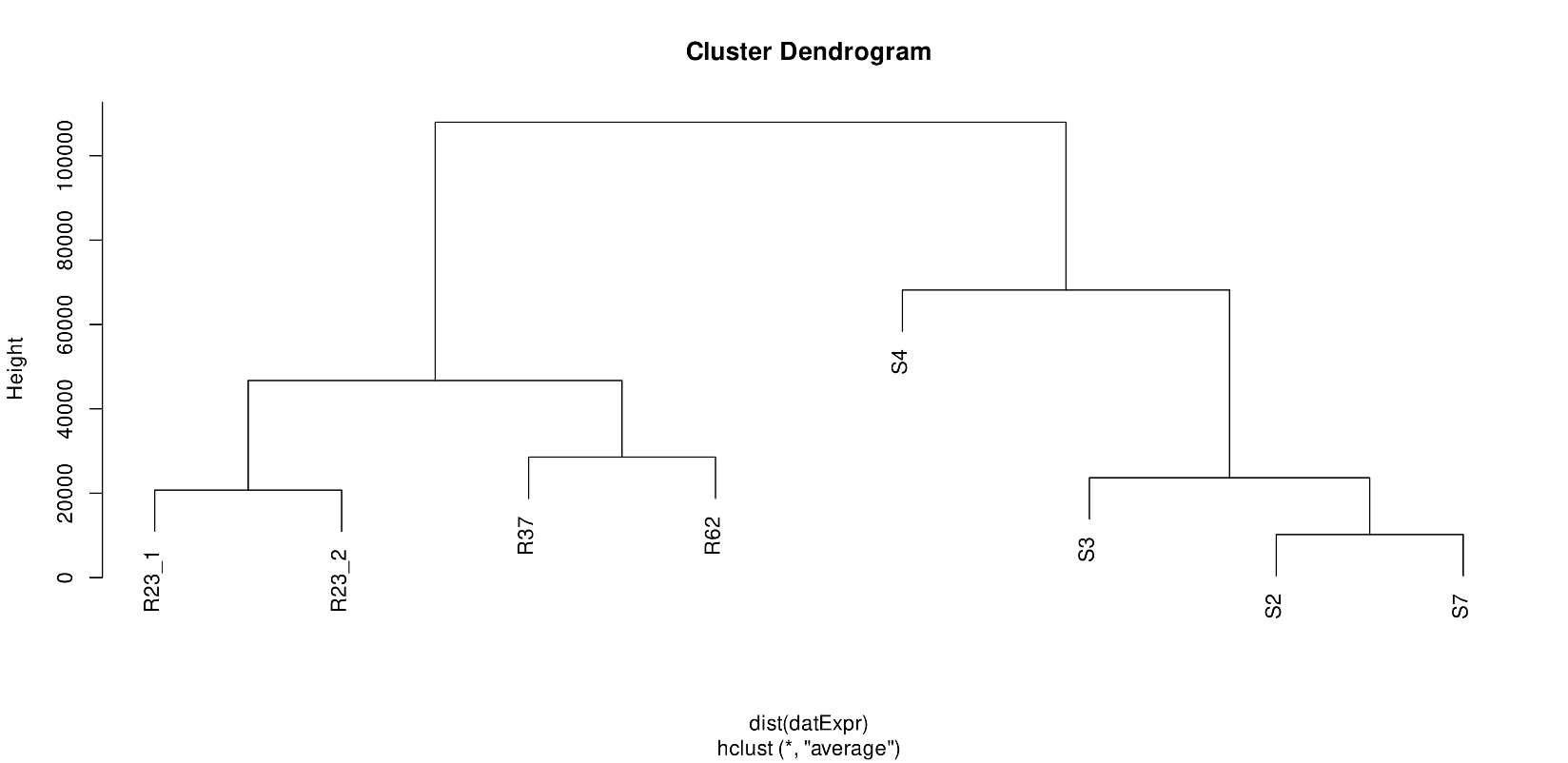


Supplementary Figure 4. Hierarchical clustering dendrogram of individuals based on gene expression data.


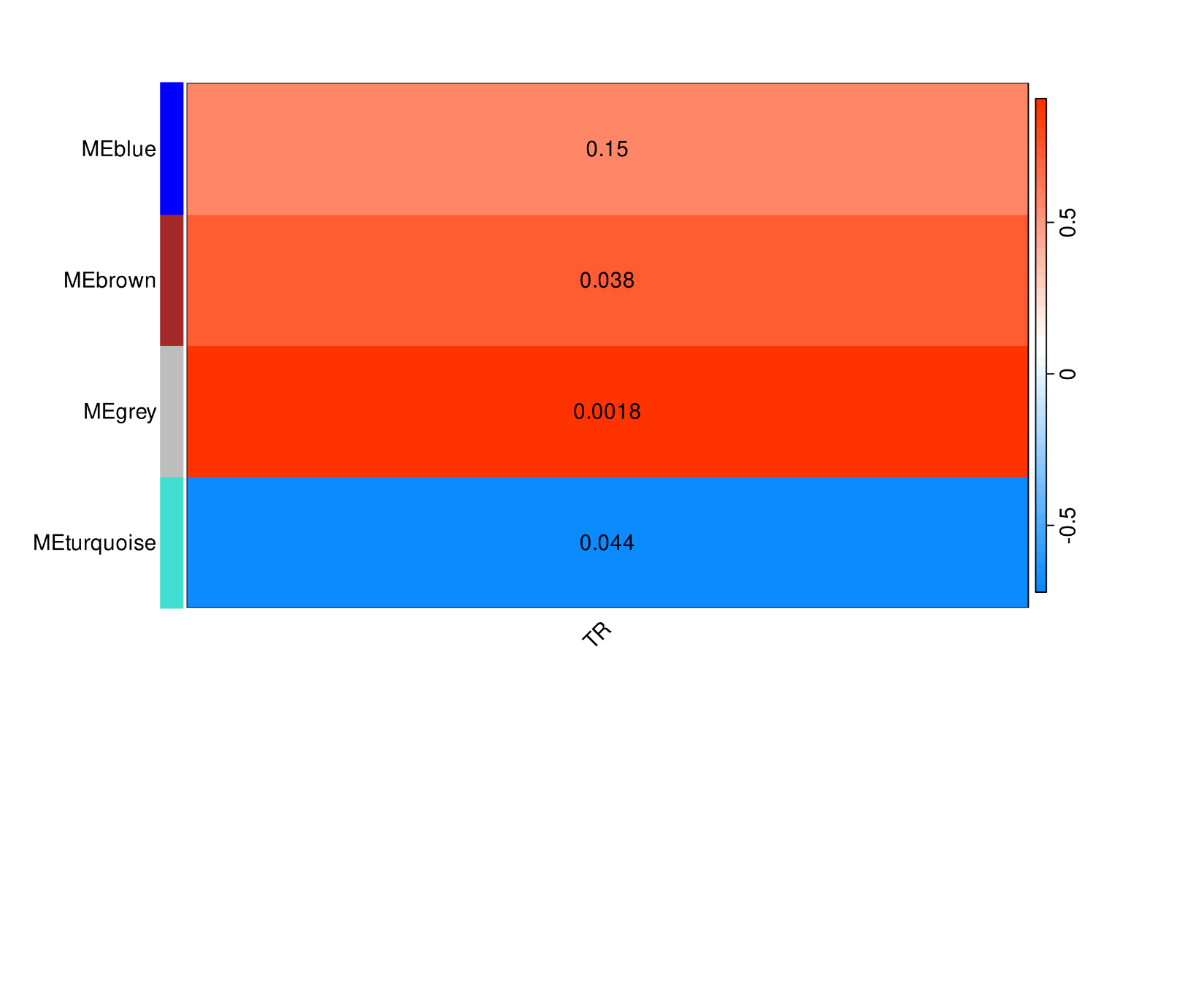


Supplementary Figure 5.Heatmap depicting correlations between WGCNA-identified gene modules (y-axis) and external traits (x-axis). Each cell displays the correlation coefficient and corresponding p-value. Blue indicates negative correlation, white indicates no correlation, and red indicates positive correlation. Strong and significant correlations suggest biologically relevant modules.
